# A randomized phase 2 trial of apatinib vs observation as maintenance treatment following first­line induction chemotherapy in extensive­ stage small cell lung cancer

**DOI:** 10.1007/s10637-019-00828-x

**Published:** 2019-08-09

**Authors:** Hao Luo, Liang Zhang, Bo Yang, Yan Feng, Yanli Xiong, Shiheng Zhang, Xuemei Li, Chengyuan Qian, Wang Dong, Nan Dai

**Affiliations:** Cancer Center, Daping Hospital & Army Medical Center of PLA, Army Medical University, Chongqing, 400042 China

**Keywords:** Apatinib, Maintenance therapy, Extensive-disease small-cell lung carcinoma, Apoptosis, Cell cycle arrest

## Abstract

*Background* The 5-year survival rate for extensive-disease small-cell lung carcinoma (ED-SCLC) is only 1%. Recently, apatinib exerted promising effects on cancer patients after failure of first-line chemotherapy. *Methods* This study enrolled 24 ED-SCLC patients to study the efficacy and toxicity of apatinib in combination with chemotherapy and maintenance therapy. The primary endpoints were overall survival (OS) and progression-free survival (PFS). The secondary endpoints included toxicity and safety. Apatinib was given 250 mg/day during the chemotherapy interval, and as maintenance therapy after 4–6 cycles until the patient progressed, died, or was intolerant to drug toxicity. The study further evaluated the cytotoxicity, cell-cycle arrest and apoptotic induction of apatinib in A549 and H446 cells. *Results* There was no difference in short-term efficacy between combined and chemotherapy groups. Long-term efficacy showed that the median PFS was 7.8 months and 4.9 months in combination and chemotherapy groups, respectively [*p* = 0.002, HR(95%CI): 0.18(0.06–0.60)]. The median OS was 12.1 months and 8.2 months in combination and chemotherapy groups, respectively [*p* = 0.023, HR(95%CI): 0.38 (0.16–0.90)]. Multivariate Cox regression analysis showed that apatinib combined with chemotherapy was an independent prognostic factor for OS and PFS. The ECOG score was an independent prognostic factor affecting OS. In vitro analysis showed that apatinib inhibited cell proliferation and caused cell-cycle arrest and apoptosis. *Conclusion* Apatinib combination/maintenance therapy showed promising efficacy and safety to extend OS/PFS in ED-SCLC and will be a potent therapeutic option in future practice. Although the scale of this study is small, further research on large sample sizes is needed.

**Electronic supplementary material:**

The online version of this article (10.1007/s10637-019-00828-x) contains supplementary material, which is available to authorized users.

## Introduction

Small-cell lung cancer (SCLC) accounts for 15–20% of the total number of lung cancers, and it is characterized by poor differentiation, rapid proliferation and high invasiveness. ED-SCLC accounts for 60–70% of all SCLC cases, and the 5-year survival rate for extensive-stage SCLC (ED-SCLC) patients is only 1% [1]. For half a century, the standard treatment for ED-SCLC includes 4–6 cycles of chemotherapy cycles with platinum and etoposide. Although patients are highly sensitive to initial treatment, many patients relapse within 6 months of first-line chemotherapy and often do not respond to subsequent chemotherapy. Previous study reported that these regimens resulted in an objective tumor response rate of 73%, and a median overall survival (OS) of 8–10 months [2–4]. The development of therapy to delay cancer progression and prolong survival after initial chemotherapy for SCLC is an unmet clinical need.

Tumor angiogenesis is an important factor affecting tumor growth [5]. Almost 80% of small cell lung cancer tissues have VEGF expression, and anti-angiogenesis studies on ED-SCLC have been tentatively explored [6]. Bevacizumab is the most widely used anti-angiogenic drug. CALGB30306 and E3501 were two early single-arm and phase 2 clinical studies that have shown to be effective and safe in SCLC [7, 8]. Subsequent studies, SALUTE and IFCT-0802, demonstrated that combined treatment with bevacizumab prolonged progression-free survival (PFS), but not OS [9, 10]. In 2015, Ready et al. reported the efficacy of sunitinib in the maintenance of ED-SCLC chemotherapy [11]. The results showed that the maintenance therapy extended PFS from 2.1 months to 3.7 months (*P* = 0.02), but was ineffective for OS [11]. Two other clinical trials of pazopanib for second-line therapy of ED-SCLC showed that PFS was extended nearly 2 months compared with placebo [12, 13]. Apatinib is a VEGFR-2 inhibitor developed in China that competes for the ATP binding site of VEGFR-2 within cells and blocks the downstream signal transduction, thereby inhibiting tumor angiogenesis [14, 15].

We designed a prospective, randomized, concurrent clinical trial to study the clinical efficacy and toxicity of apatinib in combination with chemotherapy and maintenance therapy in ED-SCLC. For reference, it is also necessary to observe the effects of apatinib on cell viability, cell cycle and apoptosis of SCLC. This study can initially explore the molecular signaling pathway, clarify the synergistic lethality of apatinib combined with chemotherapy on SCLC, and provide a new theoretical basis for guiding clinical treatment.

## Materials and methods

### Patient selection

A total of 24 patients with ED-SCLC were enrolled in the study from September 2015 to February 2018. The main inclusion criteria were: 1) diagnosis of SCLC by pathological biopsy; 2) imaging staging is extensive-stage (CT or PET-CT); 3) 18–70 years old; 4) ECOG score between 0 and 2; 5) liver, kidney and bone marrow function well; and 6) patients with brain metastasis should complete whole brain radiotherapy 4 weeks or more before the first dose and without clinical symptoms. The main exclusion criteria were: 1) ECOG score > 2; 2) estimated survival period was less than 1 month; 3) patients with other primary tumors. The design and clinical case data of this study were approved by the Ethics Committee of Daping Hospital&Army Medical Center of PLA and followed the ethical requirements.

### Treatment

This study was a prospective, randomized concurrent clinical controlled study (clinical trial information: NCT02875457), and the patients in the chemotherapy group received SCLC standard first-line chemotherapy as follows: cisplatin 80 mg/m2 or carboplatin area under the curve of 5 on day1 and etoposide 100 mg/m2 per day on days 1 to 3 every 21 days for four to six cycles. The chemotherapy regimen of the combined group was the same as the control group. Oral apatinib was given 250 mg/day during the chemotherapy interval, and as maintenance therapy after 4–6 cycles until the patient progressed, died, or was intolerant to drug toxicity. The full analysis set (FAS) included all patients who have received at least 1 cycle of treatment. The first evaluation was performed after 1 cycle (3 weeks) of administration, and the subsequent efficacy was evaluated every 2 cycles (6 weeks).

### Response and toxicity evaluation

Patients were followed up to observe the medication, efficacy and side effects. Toxicity was evaluated and graded according to the NCI CTCAE3.0 (National Cancer Institute Common Toxicity Criteria version 3.0). Tumor shrinkage was assessed according to the RECIST1.1 (Response Evaluation Criteria in Solid Tumors guidelines version 1.1). All recorded evaluations were confirmed by independent evaluators. Evaluation procedures were performed at each cycle of treatment, including physical examination, measurement of vital signs and complete blood count. The maintenance phase was evaluated monthly.

### Study endpoint and follow-up

The primary endpoints of this study were PFS and OS. The secondary endpoints included objective response rate (ORR), disease control rate (DCR), 6-months of progression-free survival rate, 12-months overall survival rate, toxicity and safety. All patients were followed up until disease progression or death. The longest follow-up period was observed as median PFS and median OS. Herein, PFS is defined as the time from the first day of treatment to the first confirmation of a patient’s disease progression or death, while the OS is the time from the first day of treatment to death for any reason, follow-up failure or follow-up deadline.

### Cell culture, reagents and cell viability assay

A549 and H446 cells were obtained from American Type Culture Collection (ATCC, Manassas, VA, USA). All cells were cultured in DMEM (Hyclone, Logan, UT, USA) containing 10% fetal calf serum (Gibco, Grand Island, NY, USA) in a 37 °C humidified incubator in 5% CO_2_. All experiments were conducted in the exponential phase of the cells.

The p-AKT antibody was obtained from Cell Signaling Technology (Beverly, MA, USA). β-actin and cyclinD1 antibody were obtained from abcam (Cambridge, MA, USA), and Bcl-2 and Bax antibody were purchased from Proteintech (Wuhan, China).

The CCK8 (Biosharp, Hefei, China) assay was used to evaluate the cell viability. Cells (5,000 cells per well) were seeded on 96-well culture plates and treated with 0, 10, 20, 40 uM apatinib (Hengrui Medicine Co. Ltd., Jiangsu, China) for 48 h. At the indicated time points, the supernatant was removed, and 100 μl of DMEM medium containing 10 μl of CCK8 was added to each well for 1.5 h at 37 °C. The absorbance was measured at 450 nm with a plate reader (Thermo Fisher Scientific, Inc., Waltham, MA, USA). The experiments were repeated three times.

### Analysis of apoptosis and cell cycle

The cells were cultured with 0, 10, 20, 40 μM of apatinib for 48 h, and apoptosis was assessed using the Annexin V-FITC kit according to the manufacturer’s instructions (BioVision, Milpitas, CA, USA). Briefly, the cells were washed twice with cold PBS, digested, collected, and resuspended in binding buffer. After the Annexin V-FITC and PI were added, the cells were incubated for 15 min at room temperature in the darkness. Then, 200 μl of binding buffer was added, and the Annexin V positive cells were analyzed using a FACS Calibur flow cytometry system (BD Biosciences, US). For cell-cycle assay, the cells were fixed with 70% ethanol at −20 °C overnight, and stained with propidium iodide. The experiments were repeated three times.

### Western blotting analysis

Briefly, H446 and H1688 cells were lysed with ice-cold RIPA buffer (Beyotime, Shanghai, China) for 30 min. Proteins were separated by SDS-polyacrylamide gel electrophoresis (SDS-PAGE) and transferred onto a PVDF membrane (Millipore). The membranes were sequentially blocked with skim milk, probed with primary antibodies, probed with HRP-conjugated secondary antibodies, and finally developed with Pierce™ ECL.

### Statistical analysis

Statistical analyses were performed using Statistical Package for Social Sciences (SPSS) software (Version 23, SPSS, Inc., Chicago, IL, USA). Data were displayed as mean ± standard deviation (SD). In addition, t-test analysis was performed. The Kaplan-Meier method was used to create the survival curve, and Cox proportional risk regression model was used to investigate the prognostic factors. *P* < 0.05 was considered to indicate a statistically significant.

## Results

### Patient characteristics

A total of 24 patients were enrolled in this study to evaluate the efficacy and safety from September 2015 to February 2018. The patients were divided into 2 group, including 12 in the combined group and 12 in the chemotherapy group. The combined group included 9 males and 3 females, with an average age of 56.4 years. Among the 12 patients, 9 patients (75%) had an ECOG (Eastern Cooperative Oncology Group) performance status score of 0 or 1, and 2 patients (16.7%) had brain metastases. The chemotherapy group included 9 males and 3 females, with an average age of 53.5 years. Among the 12 patients, 8 patients (66.7%) had an ECOG performance status of 0 or 1, and 3 patients (25%) had brain metastases. Table 1 showed the demographics and baseline characteristics of 24 patients. There was no statistically significant difference in baseline data before treatment between the two groups, including gender, age, ECOG score and number of metastatic sites (*p* > 0.05), indicating that the baseline characteristics of the two groups were basically the same and comparable.

**Table 1:** Baseline patient demographic and clinical characteristics (*n* = 24)

Characteristics	Number of patients	Apatinib combined therapy	Chemotherapy	*p*
Gender				1.000
Male	18	9	9	
Female	6	3	3	
Age (year)				0.682
<60	13	6	7	
≥60	11	6	5	
Average	54.9			
ECOG performance status				0.653
0–1	17	9	8	
2	7	3	4	
Number of the metastatic sites				0.673
0	8	3	5	
1	11	6	5	
≥2	5	3	2	
Site of metastasis				0.765
Bone	8	4	4	
Brain	5	2	3	
Liver	3	2	1	

### Comparison of short-term effects of the two groups

The response of all patients was evaluated. The objective responses were confirmed by an independent radiologist. In the combined group, one patient (8.3%) recorded as complete response (CR), 7 patients (58.3%) recorded as partial response (PR) with an ORR of 66.7%, 2 patients (16.7%) was stable disease (SD), and 2 patients (16.7%) was progressive disease (PD). On the other hand, the chemotherapy group showed that 8 patients experienced PR (66.7%), 2 patients had SD and continued for more than8 weeks, and 2 patients (16.7%) was PD. As shown in Table 2, There was no difference in short-term efficacy between the two groups.

**Table 2:** Comparison of short-term effects of the two groups

	n	CR	PR	SD	PD	ORR	DCR
Apatinib combined therapy	12	1	7	2	2	66.7%	83.3%
Chemotherapy	12		8	2	2	66.7%	83.3%

### Comparison of long-term efficacy and prognostic factors between the two groups

**Fig. 1:**
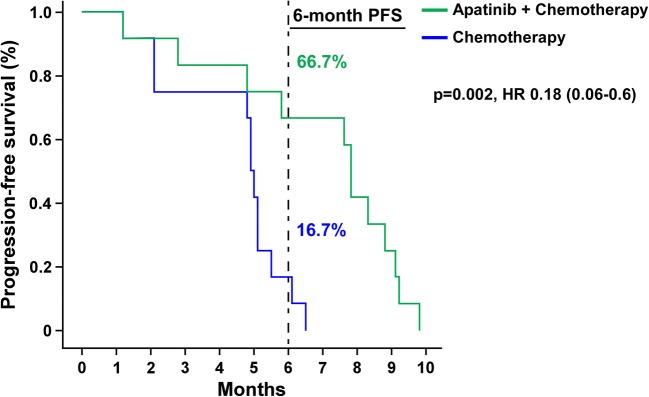
Progression-free survival in patients with ED-SCLC

**Fig. 2:**
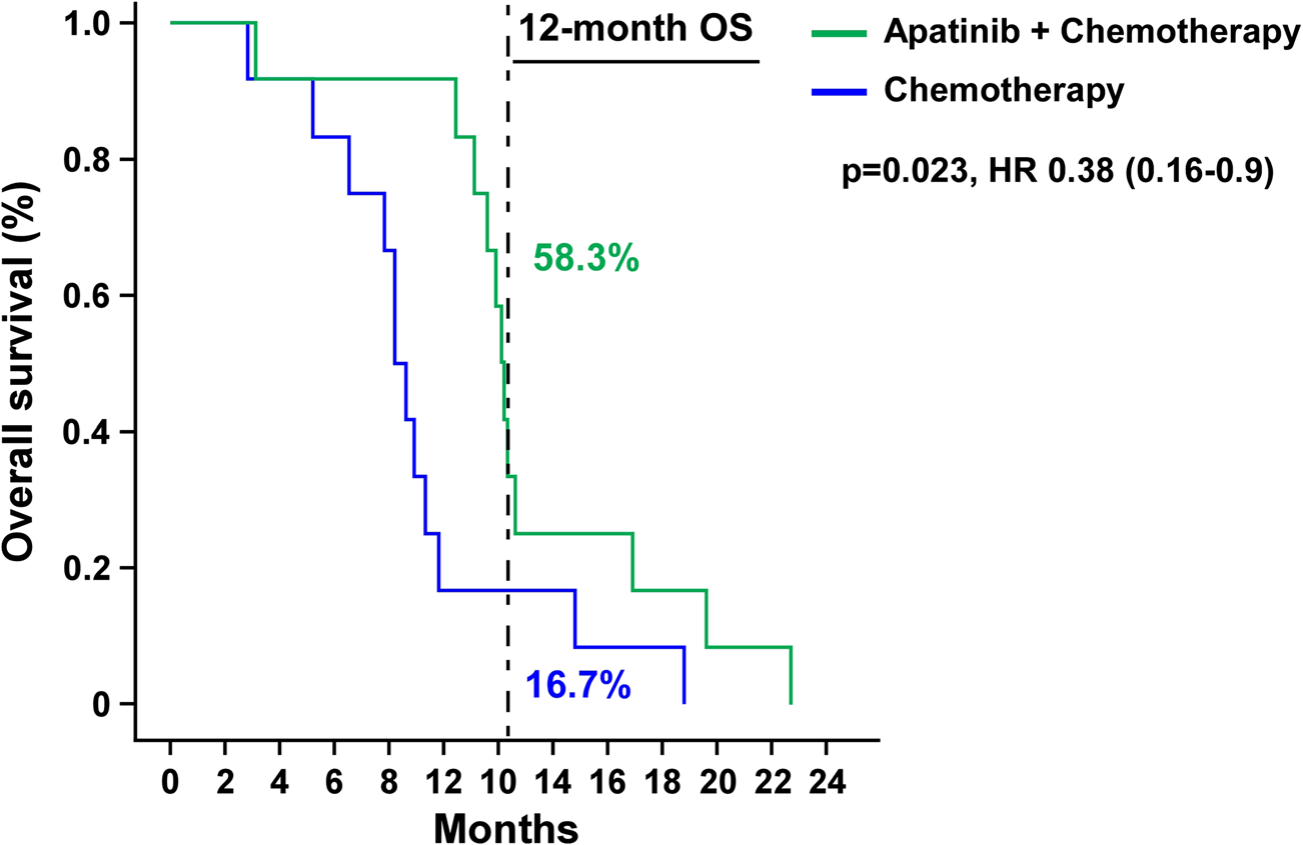
Overall survival in patients with ED-SCLC

All patients died during the follow-up period until November 30, 2018. The median PFS of the two groups was 7.8 months and 4.9 months, respectively [*p* = 0.002, HR (95%CI): 0.18 (0.06–0.60)]. In the combined group, 8 patients with PR or SD had a duration of more than 6 months, and the 6-month PFS rate was 66.7%, which was significantly better than 16.7% in the chemotherapy alone group, as shown in Fig. 1. The median OS of combined group and chemotherapy group was 12.1 months and 8.2 months, respectively (Fig. 2). Seven patients in the combined group had OS more than 12 months. The longest OS was 22.7 months, and the 12-month OS rate was 58.3% vs 16.7%. [*p* = 0.023 HR (95% CI):0.38 (0.16–0.90)]. There were significant statistical differences in PFS and OS between the two groups. Multivariate Cox regression analysis showed that apatinib combined with chemotherapy was an independent prognostic factor for OS (*p* = 0.020, HR = 0.295) and PFS (*p* = 0.005, HR = 0.152) in patients. The ECOG score (*p* = 0.014, HR = 4.370) was an independent prognostic factor affecting OS in patients (Table 3).

**Table 3:** Multivariate Cox regression analysis of PFS and OS in SCLC patients

Factor	PFS		OS	
	HR(95%CI)	*p*	HR(95%CI)	*p*
Age	0.705(0.275–1.810)	0.468	0.896(0.316–2.539)	0.836
Gender	1.852(0.697–4.930)	0.216	2.342(0.750–7.310)	0.143
ECOG	2.119(0.747–6.007)	0.158	4.370(1.345–14.198)	***0.014***
Site of metastasis	1.147(0.581–2.265)	0.693	1.173(0.609–2.259)	0.634
Apatinib combined therapy	0.152(0.041–0.563)	***0.005***	0.295(0.105–0.827)	***0.020***

Apatinib combined with chemotherapy was an independent prognostic factor for OS and PFS in patients. The ECOG score was an independent prognostic factor affecting OS in patients. ***p*** <***0.05***

### Waterfall plot of measurable lesion response

**Fig. 3:**
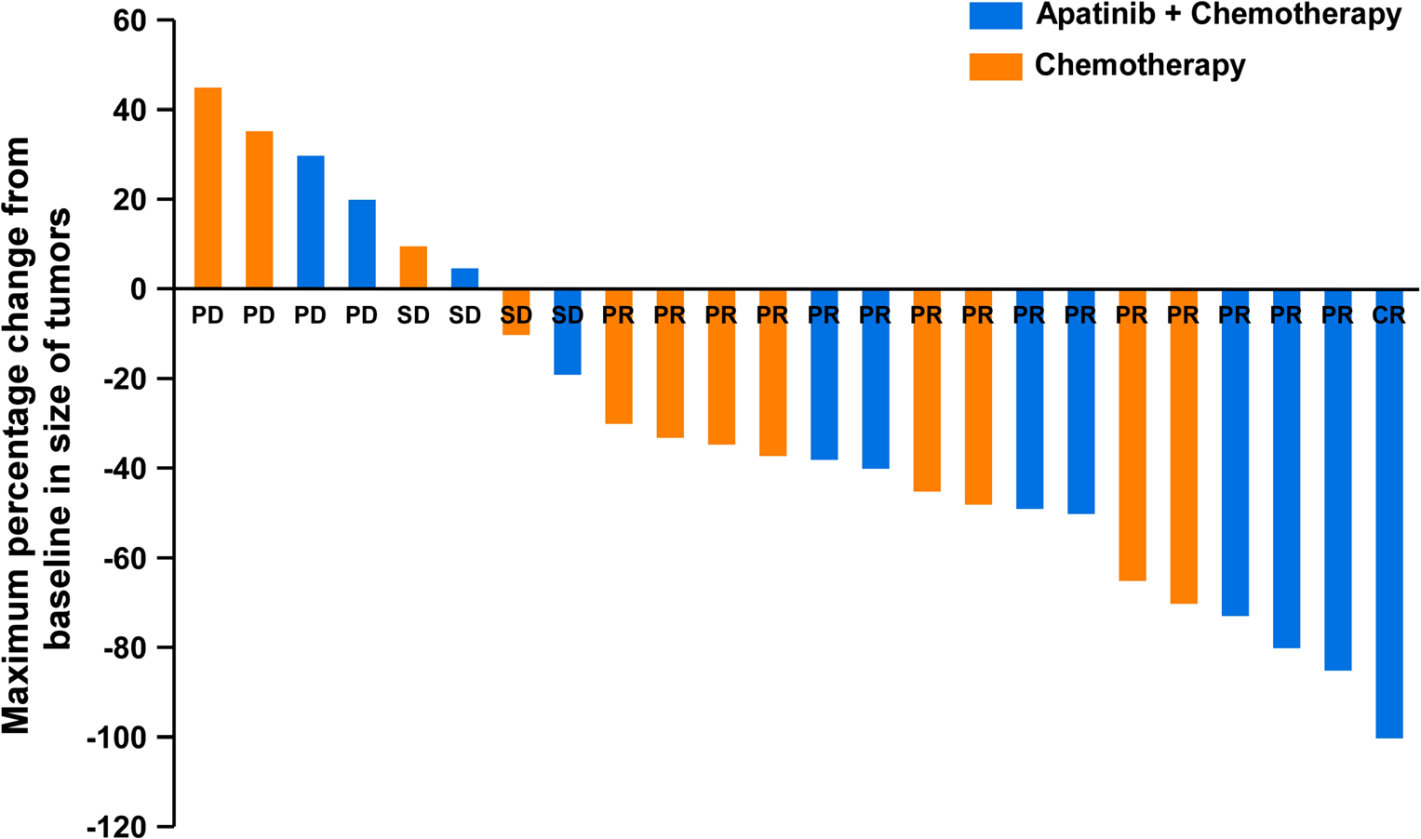
Waterfall plot of measurable lesion response

The waterfall chart of curative effect was drawn based on the clinical data of 24 patients. It showed that the lesions were reduced in 18 patients, and a total of 16 patients obtained PR. In the combined group, one patient was CR, and the maximum diameter of the tumor in 5 patients was reduced to more than 50%. The depth of remission of combined group was significantly better than that of the chemotherapy group (Fig. 3).

### Safety and toxicity

In this study, a total of 14 patients developed adverse events, and were treated symptomatically after adverse events occurred. In the combined group, there were 5 patients with grade III adverse reactions (22.7%). Among them, hand-foot skin reaction, proteinuria, fatigue and hypertension were the most important adverse events, and the incidence was higher than that in the chemotherapy group (Table 4). Recently, several studies showed that adverse events during target therapy are associated with efficacy. Thus, the Cox proportional hazards multivariate analysis was conducted to examine whether adverse events were significantly associated with PFS or OS. As shown in Table 5, adverse events including hand-foot skin reaction, hypertension, myelosuppression, proteinuria, and fatigue were not independent predictive factors of PFS and OS. This indicated that adverse events caused by apatinib-combined chemotherapy and apatinib maintenance therapy have no correlation with PFS and OS.

**Table 4:** The occurrence of adverse events

Adverse events	Apatinib combined therapy *N* = 12		Chemotherapy *N* = 12		*P*
	Type I-II	Type III-IV	Type I-II	Type III-IV	
hand-foot skin reaction	4(33.3%)	1(8.3%)	1(8.3%)	0	0.624
Hypertension	3(25.0%)	2(16.7%)	0	0	
Myelosuppression	2(9.1%)	0	3(25.0%)	1(8.3%)	0.350
proteinuria	1(8.3%)	1(8.3%)	0	0	
Oral mucosal ulcer	2(9.1%)	0	1(8.3%)	0	
Fatigue	4(33.3%)	1(8.3%)	2(16.7%)	1(8.3%)	0.673
Diarrhea	1(8.3%)	0	1(8.3%)	0	
Total case number	15	5	8	2	0.760

**Table 5:** The Cox proportional hazards multivariate analysis for PFS and OS of SCLC patients

Adverse events	Grouping	OR(95%CI) for PFS	*P value*	OR(95%CI) for OS	*P value*
Hand-foot skin reaction	Yes/no	0.797(0.148–4.287)	0.791	0.127(0.013–1.270)	0.079
Hypertension	Yes/no	1.760(0.342–9.045)	0.498	2.857(0.390–20.940)	0.302
Myelosuppression	Yes/no	1.943(0.253–14.925)	0.523	0.040(0.001–1.852)	0.100
proteinuria	Yes/no	0.142(0.010–1.919)	0.142	0.203(0.017–2.398)	0.206
Fatigue	Yes/no	0.538(0.112–2.580)	0.439	0.751(0.150–3.761)	0.728

*CI*, Confidence interval; *OR*, Odds ratio; *OS*, Overall survival; *PFS*, Progression-free survival

### Apatinib inhibits the proliferation of lung cancer cells

**Fig. 4:**
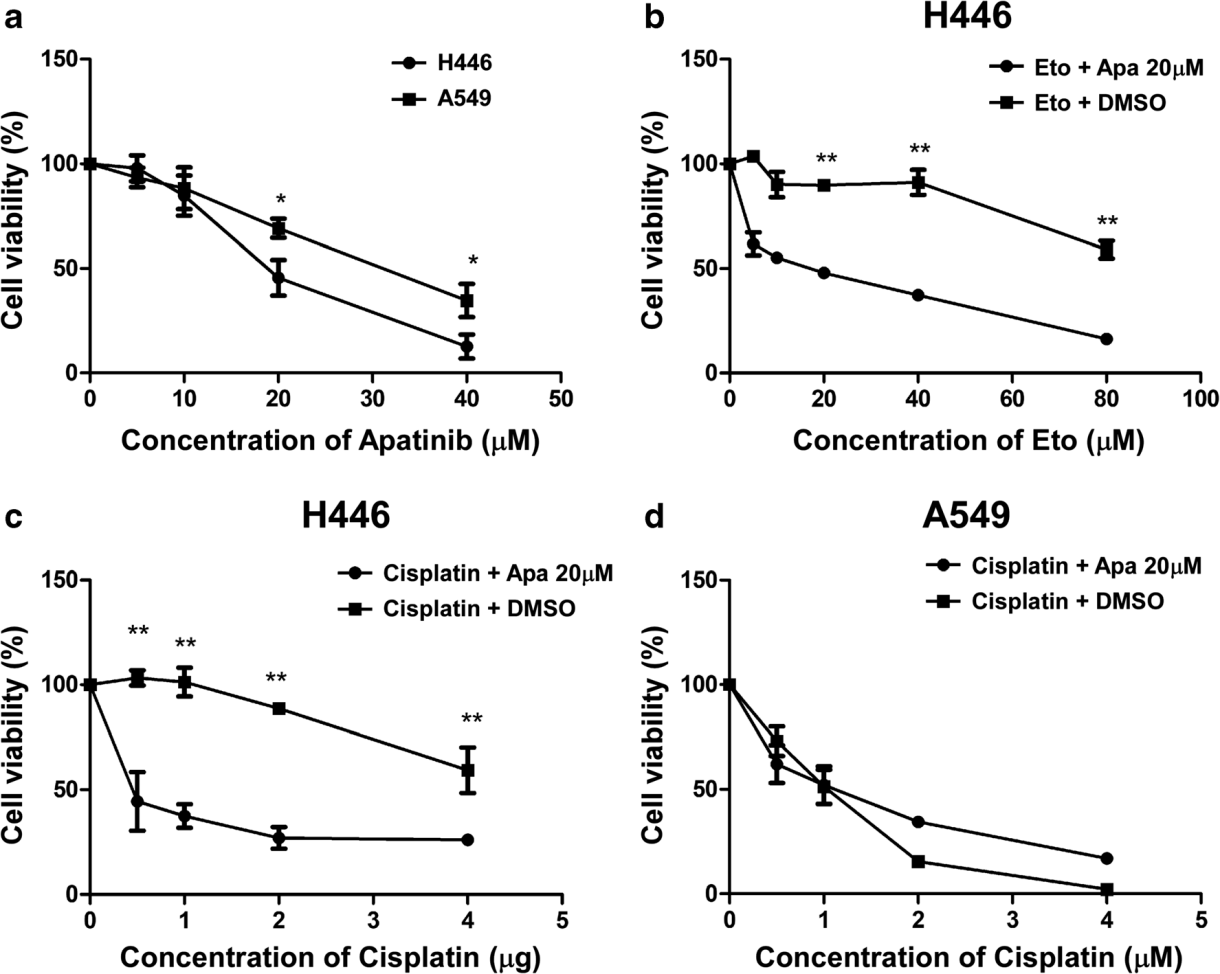
**Apatinib inhibits the proliferation of lung cancer cells and enhances the inhibitory effect of cisplatin on SCLC cells. a**: Apatinib inhibited the growth of A549 and H446 cancer cells in a concentration-dependent manner. **b**: The viability of H446 cells was measured after treatment with 20 μM apatinib or DMSO for 48 h in combination with various concentrations of Eto. **c**: The viability of H446 cells was measured after treatment with 20 μM apatinib or DMSO for 24 h in combination with various concentrations of cisplatin. **d**:The viability of A549 cells was measured after treatment with 20 μM apatinib or DMSO for 48 h in combination with various concentrations of cisplatin

To examine the effects of Apatinib in growth of lung cancer cells, the SCLC cell line H446 and non-small cell lung cancer (NSCLC) cell line A549 were used. Cells were incubated with 4 concentrations of Apatinib for 48 h, and the cell viability was determined using CCK8. As shown in Fig. 4a, the growth of both H446 and A549 were suppressed by Apatinib in a concentration-dependent manner. After 48 h treatment, the IC50 of Apatinib in H446 and A549 cells were 18.88 μM and 29.39 μM, respectively. At the same time, it was investigated whether apatinib enhances cisplatin- or etoposide-mediated proliferation inhibition. The cell viability of H446 and A549 cells after treatment with cisplatin and etoposide with or without 20 μM apatinib was determined by CCK-8 assay. The results indicated that cisplatin alone efficiently inhibited A549 cell proliferation, but moderately inhibited H446 cell proliferation. However, combined treatment with apatinib significantly enhanced cisplatin- and etoposide-mediated proliferation inhibition in H446 cells (Fig. 4b and c). Since A549 cells are original sensitive to cisplatin, no significant increase in inhibition was observed after combination with apatinib (Fig. 4d).

### Apatinib induces apoptosis and cell-cycle arrest in SCLC cells

**Fig. 5:**
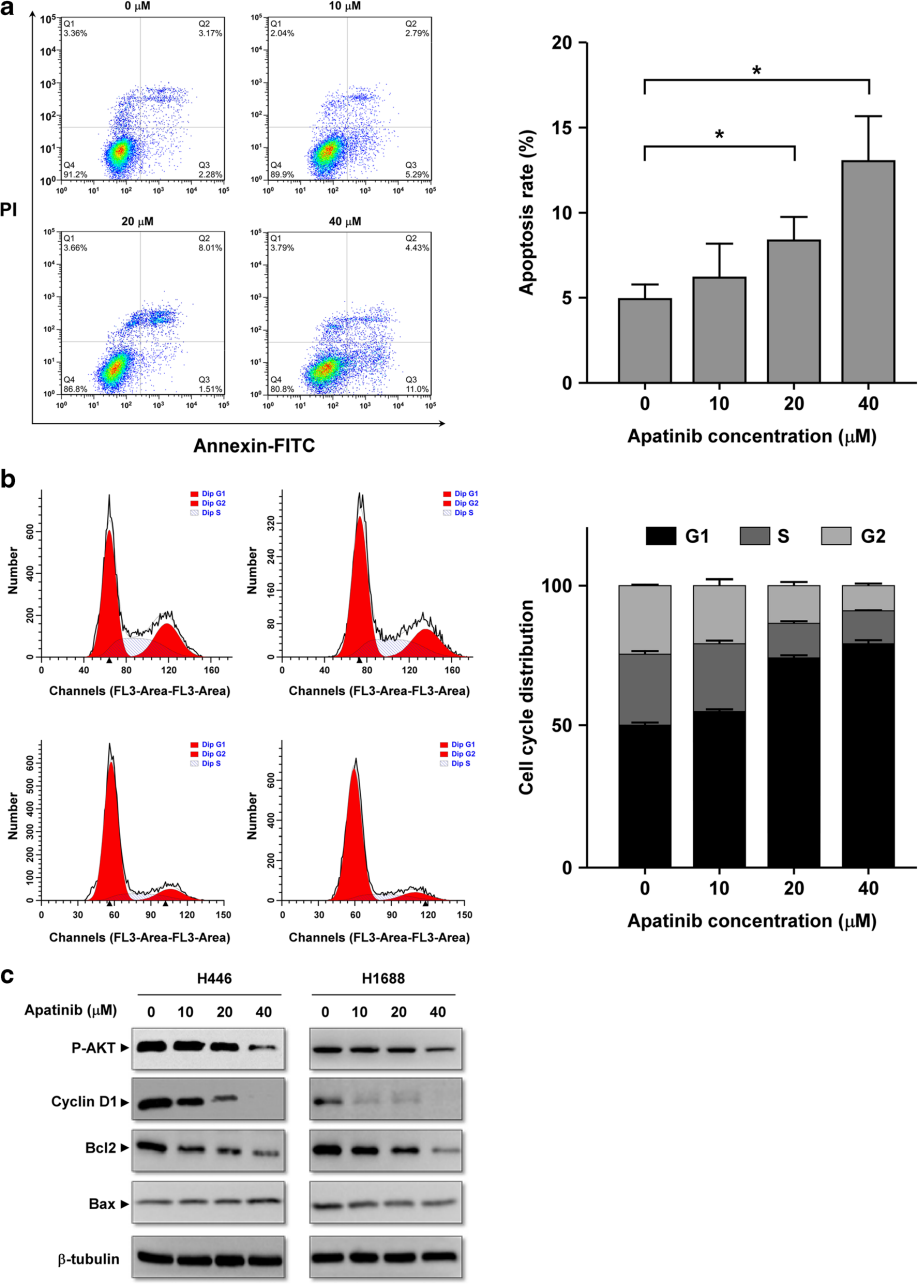
**Apatinib induces apoptosis and cell-cycle arrest in SCLC. a**: Apoptosis rates of H446 cells after incubating 0, 10, 20, or 40 μM apatinib for 48 h were determined by double staining of Annexin V and PI and quantified by flow cytometry. **b**: Apatinib caused G0/G1 cell cycle arrest in H446 cells. Cell cycle were analyzed by flow cytometry. **c**: The expressions of p-AKT, cyclin D1, Bcl-2 and Bax were determined by Western blotting analysis. Beta-tubulin was used as an internal loading control

To evaluate the apoptotic role of apatinib in SCLC cells, cell apoptosis was determined by Annexin V-FITC and propidium iodide (PI) staining and quantified by flow cytometry. Apatinib-induced cell apoptosis significantly increased when compared with the control group (Fig. 5a). To determine whether apatinib inhibited cell proliferation by inducing cell-cycle arrest, we analyzed the cell cycle distribution of apatinib-treated H446 cells. As shown in Fig. 5b, apatinib treatment induces G0/G1 cell cycle arrest and a significant decrease in the G2 and S population (Fig. 5b). After 48 h of treatment of apatinib, the key apoptosis indicators Bax and Bcl-2 increased and decreased, respectively (Fig. 5c). To elucidate the mechanisms, we determined the expression level of cyclin D1, a G0/G1-phase-related protein. As shown in Fig. 5c, the expressions of cyclin D1 decreased after treatment with apatinib. In addition, downstream targets of the VEGFR2 signaling pathway were analyzed. The level of phosphorylated AKT was reduced in apatinib-treated SCLC cells (Fig. 5c). All the data suggested that apatinib induced apoptosis and G0/G1 cell cycle arrest and inhibited the VEGFR2 signaling pathway.

## Discussion

Apatinib is a highly selective tyrosine kinase inhibitor of VEGFR2 and exerts a promising antitumoral effect in various tumors. Our clinical trial confirmed that apatinib is effective and safe in the treatment of ED-SCLC, and can prolong the duration of clinical benefit of patients. In addition, this study further demonstrated that apatinib can induce SCLC cell apoptosis and G0/G1 cell cycle arrest and affect VEGFR2 signaling pathway.

There is no standard protocol for maintenance therapy of SCLC. Due to the high toxicity of chemotherapeutic drugs, we expect anti-angiogenic agents to play an important role in maintenance therapy. Based on the expression of high microvessel density and vascular endothelial growth factor in nearly 80% of SCLC cases, angiogenesis is critical in SCLC [16]. In 2007, a phase 2 clinical trial of thalidomide as a maintenance therapy for SD-SCLC indicated that median survival from time of initiation of induction chemotherapy was 12.8 months (95% CI: 10.1–15.8 months) and 1-year survival of 51.7% (95% CI: 32.5–67.9%). When administrated as maintenance therapy for ED-SCLC after induction chemotherapy, 200 mg of thalidomide per day was well tolerated. However, the subsequent phase III clinical study found that thalidomide combined with chemotherapy shortened patient survival and increased the risk of thrombosis [17]. Moreover, studies on the application of imatinib and vandetanib in SCLC found that the addition of these drugs cannot bring survival benefits [18]. Another phase 2 study of sunitinib found that although the benefits of OS were not achieved, but the use of sunitinib extended PFS (median PFS was 3.7 months vs 2.8 months and median OS was 9 months vs 6.9 months) [11]. There are also reports of immunotherapy for SCLC. CheckMate 331 study found that Nivolumab did not show superior efficacy in SCLC patients who have relapsed after first-line chemotherapy. But IMpower 133 study found combined atezolizumab for the first-line chemotherapy of ED-SCLC showed a significantly longer OS and PFS than chemotherapy alone [19]. Other studies found that the efficacy of apatinib in the treatment of ED-SCLC after two or more chemotherapy failures is effective and safe [20, 21].

The results of this clinical study showed that apatinib combined with EP regimen significantly improved OS and PFS in ED-SCLC patients compared with chemotherapy alone. The median PFS was 7.8 months and 4.9 months, respectively [*p* = 0.002, HR (95% CI): 0.18 (0.06–0.60)]. The median OS of the two groups was 12.1 months and 8.2 months, respectively. The respective OS rate at 12 months was 58.3% vs 16.7% [*p* = 0.023 HR (95% CI): 0.38 (0.16–0.90)]. Statistical analysis suggested that the PFS and OS of two groups has significant statistical differences. In this study, 14 patients suffered from various adverse events, and the incidence of adverse events was 58.3%. Among them, 5 patients (33.3%) in the combined group had grade III or above adverse reactions, including hand-foot skin reactions, proteinuria and hypertension. In consistent to our study, Shi et al. [22]. found that in the treatment of advanced NSCLC with apatinib, the incidence of adverse events was higher in the apatinib group, mainly proteinuria, hypertension and hand-foot syndromes. Multivariate Cox regression analysis showed that apatinib combined with chemotherapy was an independent prognostic factor for OS and PFS in patients. This suggests that apatinib may be a clinical option for combination chemotherapy or single-agent maintenance therapy for patients with good clinical physical strength, low tumor burden, and lack of effective treatment and maintenance. Apatinib can prolong the patient’s sustained clinical benefit, but current evidence is insufficient. Anti-angiogenic drugs are more at risk for bleeding and hypertension, and the benefits and risks need to be fully weighed. In our in vitro study, apatinib significantly enhanced cisplatin- or etoposide-mediated proliferation inhibition in H446 cells when compared with cells treated with cisplatin alone. In addition, our data showed that apatinib can induce cell apoptosis and G0/G1cell cycle arrest and affect VEGFR2 signaling pathway. Liu et al. [23]. reported that apatinib could promote autophagy and apoptosis through VEGFR2/STAT3/BCL-2 signaling in osteosarcoma. The same study by Peng et al. [24] indicated that apatinib could inhibit VEGF signaling and promote apoptosis of intrahepatic cholangiocarcinoma.

Adverse events are often observed in cancer patients undergoing therapeutic treatment. Recently, several studies showed that adverse events during target therapy were associated with efficacy, such as cetuximab and panitumumab in colorectal cancer [25], cetuximab in advanced head and neck cancer [26], sunitinib and sorafenib in metastatic renal cell carcinoma [27], and sunitinib in metastatic renal cell carcinoma [28]. In this study, however, adverse events were not associated with efficacy in ED-SCLC patients treated with apatinib-combined and maintenance therapy. These ED-SCLC patients were less likely to develop these adverse effects. Since the poor treatment outcome was not related to adverse events, in addition to active supportive care in the future, a threshold for increasing the therapeutic dose may be set. However, it must be understood that increasing the therapeutic dose of apatinib may be a double-edge sword because it may simultaneously increase the efficacy and worse OS/PFS. Thus, we will further explore and strengthen this part in future studies.

Studies have shown that angiogenesis is a key mechanism of tumor growth and an important step in tumor progression, invasion and metastasis [29]. VEGF/VEGFR2 is an important signaling pathway for tumor angiogenesis, in which VEGF has the ability to enhance tumor invasion and survival, and can play the role of cancer stem cells. On the other hand, VEGF also has the function of recruiting regulatory T cells, which can inhibit the body’s anti-tumor immune response. VEGFR2 is a transmembrane protein, and VEGF specifically binds to the extracellular domain of VEGFR2 to activate mitogen-activated protein kinase (MAPK), phosphatidylinositol 3-kinase (PI3K), protein kinase C (PKC), focal adhesion kinase (FAK) and other downstream signaling pathways. Activation including cell proliferation, migration, permeability and survival plays a primary role in angiogenesis and production. Therefore, anti-angiogenic targeted drugs are the hotspots in recent years for the treatment of NSCLC. Apatinib is highly selective tyrosine kinase inhibitor of VEGFR2. When targeting to VEGFR-2, the tyrosine kinase activity of the cells was inhibited, resulting in the inhibition of VEGF/VEGFR-2 signaling pathway and subsequent inhibition of tumor angiogenesis and tumor progression. The process of inhibiting tumor angiogenesis can effectively reduce tumor progression and metastasis [30].

In the past few years, lung cancer tumors with very poor prognosis have only achieved minor therapeutic success. The main reason for most chemotherapy failures is the development of chemoresistance. Most lung cancer patients will eventually develop resistance to the chemotherapeutic agents which they exposed to, even with good initial response. In addition to active efflux of the chemotherapeutic agent from tumor cells, hypoxic tumor microenvironment and hypoxia-mediated upregulation of VEGF play an important role in the hypervascularization, forming new blood vessels to supply nutrient and oxygen for tumor progression and recurrence. Since many therapeutic drugs cannot increase the overall survival after the failure of chemotherapy, VEGF-targeting by apatinib can be combined with traditional treatment modalities to ensure maximum effectiveness. In order to reduce chemoresistance, continuous use of low doses of apatinib may inhibit VEGF-mediated angiogenesis (the basic function of apatinib). In particular, apatinib was able to prevent multidrug resistance (MDR) of cancer cells against other conventional chemotherapeutic drugs by inhibiting ABCB1 and ABCG2-mediated drug export [31]. Increased accumulation of doxorubicin was found in the apatinib-treated MDR cells. In addition, the effect of apatinib maintenance on prolonging OS may be due to the promotion of tumor cell apoptosis and cell cycle arrest (Figs. 4 and 5). Thus, it is the possible reason that apatinib maintenance therapy combined with chemotherapy can lead to a significant prolongation of overall survival and progression-free survival in ED-SCLC patients.

There was no breakthrough in patients with ED-SCLC, with a median OS of 8–10 months. The main reason may be that ED-SCLC produces strong drug resistance and tumor immune microenvironment, when it progresses after first-line treatment, resulting in extremely poor efficacy of second-line treatment. Although apatinib showed advantage in the tumor MDR, the problem of tumor immune microenvironment was not improved. Recently, checkpoint blockade immunotherapy received promising attention in the treatment for ED-SCLC. Although phase II [32] and III [33] trial showed no significant differences between the control and ipilimumab (anti-CTLA4) groups, PD-L1 antibody (nivolumab, pembrolizumab, atezolizumab) showed its safety and clinical efficacy [34–37]. Furthermore, recent clinical studies demonstrated that combined treatment with pembrolizumab and chemotherapy showed significantly benefit in advanced NSCLC [38, 39], advanced or metastatic platinum-refractory urothelial cancer [40], and advanced gastric or gastroesophageal junction adenocarcinoma [41]. Considering the results of KEYNOTE-024 and 189 [42, 43], the introduction of immunotherapy as a first-line therapy may have a beneficial long-term effect on the results. The future of ED-SCLC treatment may combine PD-L1 (e.g. pembrolizumab) and chemotherapy to maximize efficacy, and followed by apatinib maintenance therapy to eliminate tumor MDR effect.

In conclusion, our study showed that apatinib has significant efficacy and high safety in the treatment of ED-SCLC. It can clinically extend the patient’s sustained duration, and it can be further studied and applied in clinical practice. Although the sample size of this study is small, further studies with larger sample size are needed.

## Electronic supplementary material

ESM 1 (DOCX 119 kb)
