# Supplementary material for: A randomized phase 2 trial of apatinib vs observation as maintenance treatment following first­line induction chemotherapy in extensive­ stage small cell lung cancer
Source: Invest New Drugs. 2019 Aug 9;38(1):148–59. doi: 10.1007/s10637-019-00828-x (PMC6985106; doi:10.1007/s10637-019-00828-x)

**Supplementary information:** Schematic diagram of maintenance therapy with apatinib combined with chemotherapy in patients with ED-SCLC


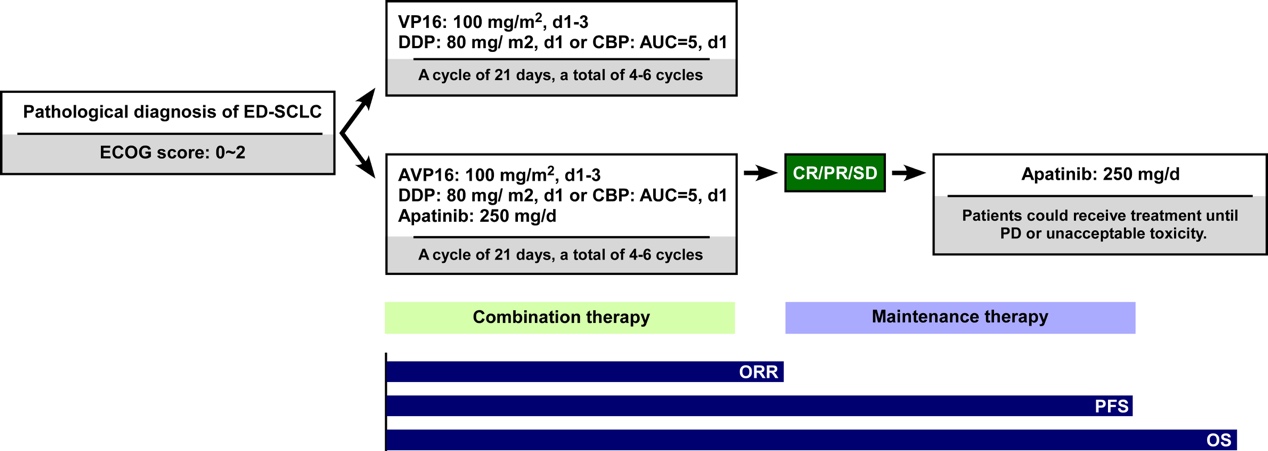

Supplement: Supplementary file 1 — (DOCX 119 kb) [file 10637_2019_828_MOESM1_ESM.docx]
